# Differential microRNA expression in the peripheral blood from human patients with COVID‐19

**DOI:** 10.1002/jcla.23590

**Published:** 2020-09-22

**Authors:** Caixia Li, Xiao Hu, Leilei Li, Jin‐hui Li

**Affiliations:** ^1^ Department of General practice School of Medicine The Fourth Affiliated Hospital Zhejiang University Yiwu China; ^2^ Department of Anesthesiology The First Affiliated Hospital School of Medicine Zhejiang University Hangzhou China; ^3^ Department of Laboratory Medicine School of Medicine The Fourth Affiliated Hospital Zhejiang University Yiwu China; ^4^ Department of Operating Room School of Medicine The Fourth Affiliated Hospital Zhejiang University Yiwu China; ^5^ Department of Rehabilitation Medicine School of Medicine The Fourth Affiliated Hospital Zhejiang University Yiwu China; ^6^ Department of Rehabilitation and Traditional Chinese Medicine The Second Affiliated Hospital of Zhejiang University School of Medicine Hangzhou China

**Keywords:** microRNA, high‐throughput sequencing, Bioinformatics analysis, COVID‐19

## Abstract

**Introduction:**

The coronavirus disease (COVID‐19) is caused by severe acute respiratory syndrome coronavirus 2 (SARS‐CoV‐2), which play important roles in regulating gene expression and are also considered as essential modulators during viral infection. The aim of this study was to elucidate the differential expression of miRNAs in COVID‐19.

**Methods:**

The total RNA was extracted and purified from the peripheral blood of ten patients with COVID‐19 and four healthy donors. The expression levels of various miRNAs were detected by high‐throughput sequencing, and correlation analysis was performed on the target genes that are primed by miRNAs.

**Key findings:**

Compared with the healthy controls, 35 miRNAs were upregulated and 38 miRNAs were downregulated in the human patients with COVID‐19. The top 10 genes were listed below: hsa‐miR‐16‐2‐3P,hsa‐miR‐5695,hsa‐miR‐10399‐3P,hsa‐miR‐6501‐5P,hsa‐miR‐361‐3P,hsa‐miR‐361‐3p, hsa‐miR‐4659a‐3p, hsa‐miR‐142‐5p, hsa‐miR‐4685‐3p, hsa‐miR‐454‐5p, and hsa‐miR‐30c‐5p. The 10 genes with the greatest reduction were listed below: hsa‐miR‐183‐5p, hsa‐miR‐627‐5p, hsa‐miR‐941, hsa‐miR‐21‐5p, hsa‐miR‐20a‐5p, hsa‐miR‐146b‐5p, hsa‐miR‐454‐3p, hsa‐miR‐18a‐5p, hsa‐miR‐340‐5p, and hsa‐miR‐17‐5p. Remarkably, miR‐16‐2‐3p was the most upregulated miRNA, with a 1.6‐fold change compared to that of the controls. Moreover, the expression of miR‐6501‐5p and miR‐618 was 1.5‐fold higher in the COVID‐19 patients than in the healthy donors. Meanwhile, miR‐627‐5p was the most downregulated miRNA, with a 2.3‐fold change compared to that of the controls. The expression of other miRNAs (miR‐183‐5p, miR‐627‐5p, and miR‐144‐3p) was reduced by more than 1.3‐fold compared to that of the healthy donors. Cluster analysis revealed that all of the differentially expressed miRNA target genes were clustered by their regulation of cellular components, molecular functions, and biological processes. Importantly, peptidases, protein kinases, and the ubiquitin system were shown to be the highest enrichment categories by enrichment analysis.

**Conclusions:**

The differential miRNA expression found in COVID‐19 patients may regulate the immune responses and viral replication during viral infection.

## Graphical abstract

Extract periperal blood cells from COVID‐19 patients, and micRNAs were detected by high‐throughput sequencing using illumina HiseqX Ten machine to fint the differential expression of miRNAs in COVID‐19.

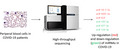

## 1 INTRODUCTION

In December 2019, severe acute respiratory syndrome coronavirus 2 (SARS‐CoV‐2) infection was discovered in human patients with previously unidentified respiratory‐related syndromes in Wuhan, China. Later, the disease caused by this virus was named as COVID‐19 by the World Health Organization.^1^, ^2^, ^3^ COVID‐19 has caused considerable impacts on the global economy and public health. After the initial outbreak in Wuhan, it has reached many countries throughout the world to cause a global pandemic since March 2020.^4^, ^5^, ^6^, ^7^ Classified as a member of the family Coronaviridae and the order Nidovirales, coronaviruses are a group of enveloped positive‐stranded RNA viruses that can infect many animals, including humans, bats, pangolins, etc. Genomic studies on the viral sequence of SARS‐CoV‐2 suggest that that this virus shares 89% nucleotide identity with bat SARS‐like‐CoVZXC21 and 82% nucleotide identity with human SARS‐CoV‐1.^8^ However, the external subdomain of the receptor binding domain of SARS‐CoV‐2 shares less than 50% amino acid identity with other SARS‐related coronaviruses.^8^, ^9^

MicroRNAs (miRNAs) are highly conserved and short non‐coding RNA molecules that usually contain about 18‐25 nucleotides. They are widely found in plants, animals, and some viruses. Existing evidence suggests that miRNAs play critical roles in regulating gene expression by targeting the mRNAs of protein‐coding genes.^10^, ^11^ The dysregulation of miRNAs has been shown to be associated with disease severity and therapeutic outcomes by different treatments. Moreover, altered expression levels of miRNAs are also associated with various diseases.^10^, ^12^ Strikingly, recent studies have shown that miRNAs, as vital modulators, are also involved in the regulation of viral infection and host defense.^13^, ^14^, ^15^ For instance, previous data suggest that specific miRNAs in hepatitis B virus are associated with liver disease and that Epstein‐Barr virus‐encoded miRNAs collaborate to assist in host immune escape.^13^, ^14^, ^15^ Therefore, existing results indicate that viral infection can affect host homeostasis by regulating miRNA expression and that altered expression of miRNAs can cause other genes to regulate the host immune response to viral infection. MicroRNAs effect on virus replication on its pathogenesis and they have recently emerged as important modulators of viral infections and will play an important role in the treatment of viral enfections in near future.^16^

In this study, to better understand the miRNA expression pattern in peripheral blood collected from human COVID‐19 patients and healthy donors, high‐throughput sequencing, and bioinformatics analysis were employed. To the best of our knowledge, this study is the first to investigate the miRNA expression profiles in COVID‐19 patients.

## 2 MATERIALS AND METHODS

### 2.1 Characteristics of the subjects

The patients were divided into two groups: group A (COVID‐19 group, n = 10) and group B (control group, n = 4). Both groups were enrolled at the Fourth Affiliated Hospital, College of Medicine, Zhejiang University between February and March 2020. To protect the privacy of the patients, the ten patients diagnosed and confirmed with COVID‐19 were named as X1–X10, and the four healthy donors were named as X11–X14. Table 1 shows the characteristics of the patients enrolled in this study. All patients with mild or moderate symptoms all were included in our study.

**Table 1:** Sex and age of the enrolled patients in this study

Group	Sex (male/female)	Age (years)
COVID‐19 (group A)	4/6	44.90 ± 19.94
Control group (group B)	2/2	44.75 ± 11.84

### 2.2 Patient management

All patients were treated with antiviral reagents and medical support after diagnosis. All specimens were collected within one week. Peripheral blood and stool specimens from patients were collected and stored in a −80 degree refrigerator for testing.

### 2.3 RNA extraction

The whole blood (2 mL) was extracted from all donors by using BD PAXgene blood RNA tubes (BD, cat. no. 762165). The blood was then mixed up and down 8–10 times. After that, the PAXgene tubes were incubated at room temperature for at least 2 h to ensure that the blood cells were completely dissolved and stored at −80°C. Next, RNA extraction was performed by using a Qiagen PAXgene Blood miRNA kit (Qiagen, cat. no. 763134). Briefly, the total RNA samples (1 µg) were reacted with a 3’ adaptor and combined with reverse transcription primers. 5’ adaptors were connected after the previous reaction. Finally, reverse transcription was performed on both sides of the joint, followed by polymerase chain reaction (PCR) for some of the products.

### 2.4 High‐throughput sequencing

After the PCR, the product was loaded onto a 6% polyacrylamide gel and electrophoresed. The purified product was used for the construction of the final library. The sequencing platform was an Illumina HiseqX Ten, and the sequencing model was PE75.

### 2.5 Bioinformatics analysis

The quality of the raw data was evaluated, and filtered reads were compared to the genome using miRdeep2 (https://www.mdc-berlin.de/8551903/en/). The results were normalized as reads per million. The miRNA target genes were predicted with miRanda (http://www.microrna.org/microrna/home.do). Finally, differential expression analysis of miRNAs was performed. Gene ontology (GO, http://www.geneontology.org/) was used for enrichment analysis. Differentially expressed genes were classified according to the cellular component, molecular function, and biological process.

### 2.6 Statistical analysis

Continuous variables were expressed as the median (interquartile range), and data were compared by the Mann‐Whitney U test. The data were analyzed by Pearson’s correlation analysis and principal component analysis. Based on the expression levels of existing known and newly predicted miRNAs, the package DESeq2 of R software was used to screen the differentially expressed miRNAs. A *P*‐value <.05 and log2 (fold change) ≠ 0 indicated differentially expressed miRNAs between the two groups. The miRNAs with log2 (fold change)> 0 were labeled as upregulated miRNAs (Up), while miRNAs with log2 (fold change) < 0 were labeled as downregulated miRNAs (Down).

## 3 RESULTS

### 3.1 Quality check of the raw data

A small number of reads from the original sequencing data had artificial sequences such as sequencing primers and connectors. To ensure the accuracy of subsequent analyses, the data were filtered to remove low‐quality reads affecting the quality of the data. The results are shown in Table 2.

**Table 2:** The quality filtration of raw data

Sample	Raw_data_reads	clean_data_reads	clean_data_bases	clean_data_q30_rate
X1	10583236	9931496	208139966	0.979974
X2	15072311	13963383	293674233	0.984484
X3	10681273	9809574	209638833	0.977304
X4	10126869	9392398	196711582	0.984452
X5	9584324	8802594	186714478	0.984216
X6	12839496	11968981	249009803	0.985468
X7	13665638	12781167	269557963	0.983232
X8	16402692	15256615	320507665	0.984812
X9	13953013	13002652	278028345	0.983793
X10	12649986	11742479	244667004	0.983885
X11	12869132	12290801	262422289	0.974062
X12	14015874	12997476	273625028	0.984403
X13	10380525	8972776	192983484	0.983388
X14	13270590	12518179	266030852	0.983940

### 3.2 Differential miRNA expression profiling

**Figure 1:**
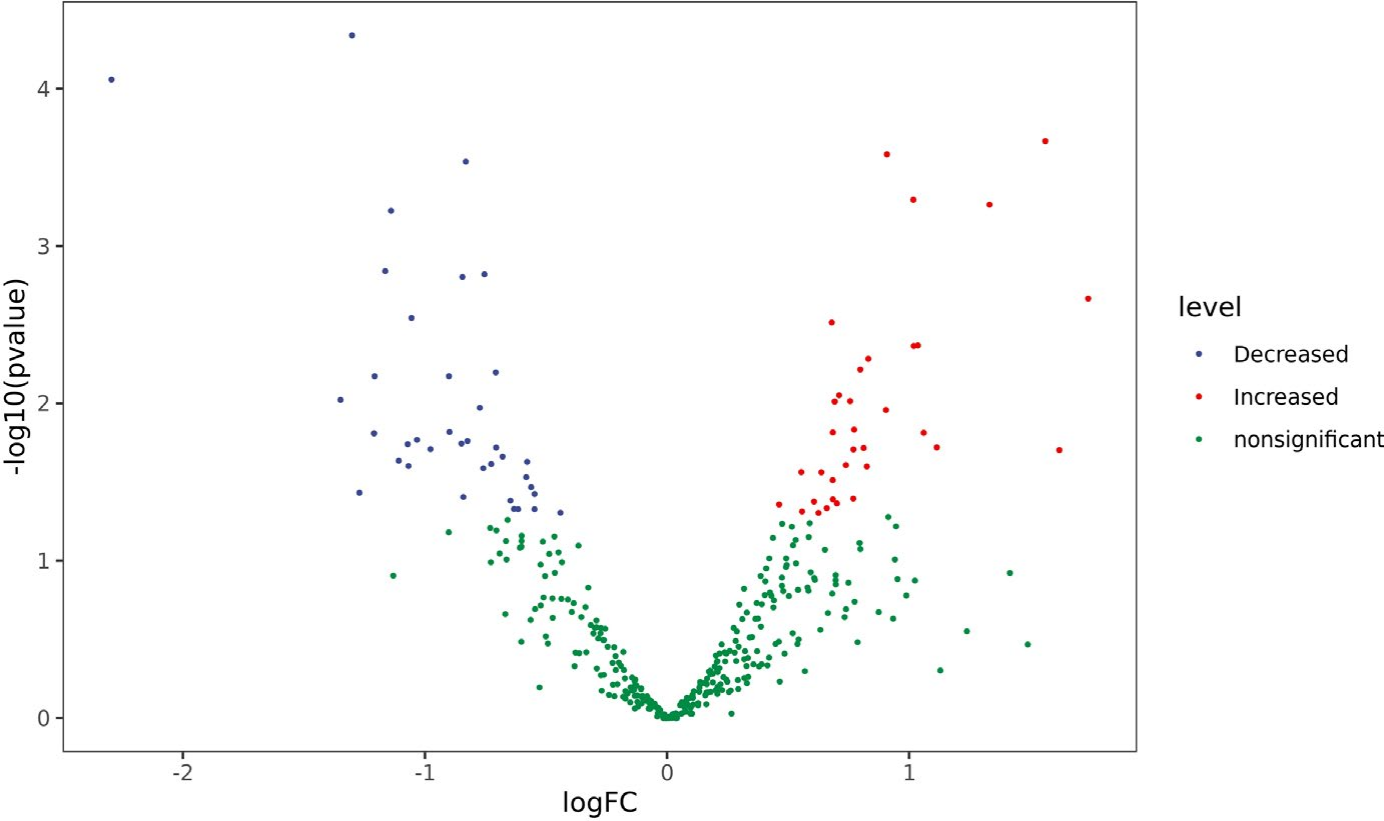
Differential miRNA expression between COVID‐19 patients and healthy donors. Each point in the figure represents a gene, and the abscissa represents the logarithmic value logFC of the multiple differences of gene expression between the two groups. The ordinate represents the negative pair value of the p‐value of the change in gene expression. The greater the absolute value of the abscissa, the greater the difference of expression between the two groups. The larger the ordinate value, the more significant the difference in expression. Genes with significant differences are represented by red and blue dots, and genes without significant differences are represented by green dots

**Figure 2:**
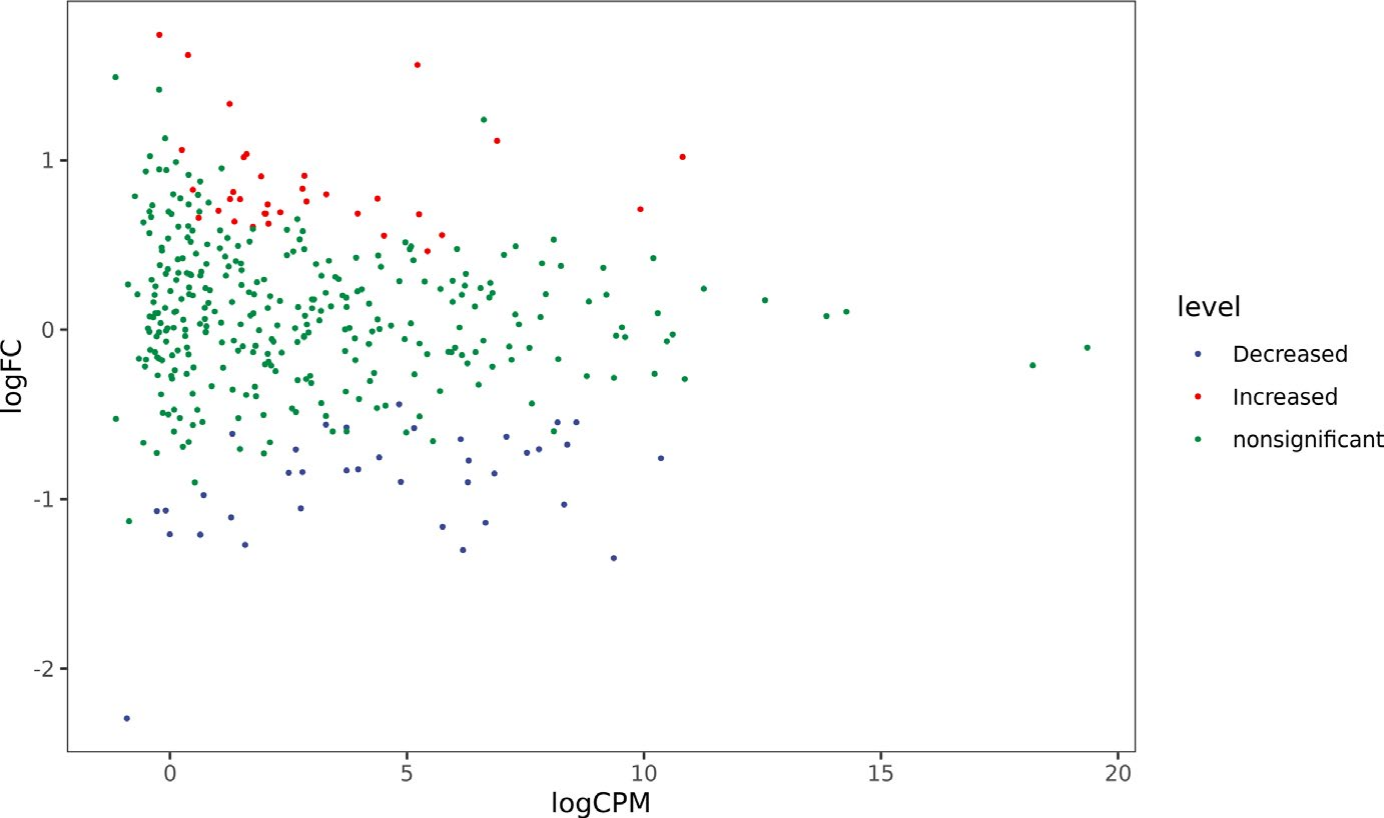
Differential miRNA expression between COVID‐19 patients and healthy donors. Genes with significant differences are represented by red and blue dots, and genes without significant differences are represented by green dots

A total of 73/390 human miRNAs were differentially expressed between the COVID‐19 group (group A) and the control group (group B) (Figures 1 and 2). A total of 35 human miRNAs were significantly upregulated, while 38 human miRNAs were significantly downregulated (*P* < .05). Furthermore, the expression of miR‐16‐2‐3p, miR‐6501‐5p, and miR‐618 was at least 1.5‐fold higher in the COVID‐19 patients than in the control group. Importantly, miR‐16‐2‐3p was the most upregulated miRNA, with a 1.6‐fold change compared to that of the control group. Whereas miR‐627‐5p was the most downregulated miRNA, with a 2.3‐fold reduction compared to that of the control group. In addition, the expression of miR‐183‐5p, miR‐627‐5p, and miR‐144‐3p was reduced by more than 1.3‐fold compared to that of the healthy donors (Table 3).

**Table 3:** miRNAs implicated in COVID‐19

miRNA name	Fold change	Regulation direction	*P*‐value
miR‐16‐2‐3p	1.56	up	<.001
miR‐6501‐5p	1.74	up	.002
miR‐618	1.62	up	.02
miR‐183‐5p	1.30	down	<.001
miR‐627‐5p	2.29	down	<.001
miR‐144‐3p	1.35	down	.01

### 3.3 Cluster analysis of differential miRNA expression

**Figure 3:**
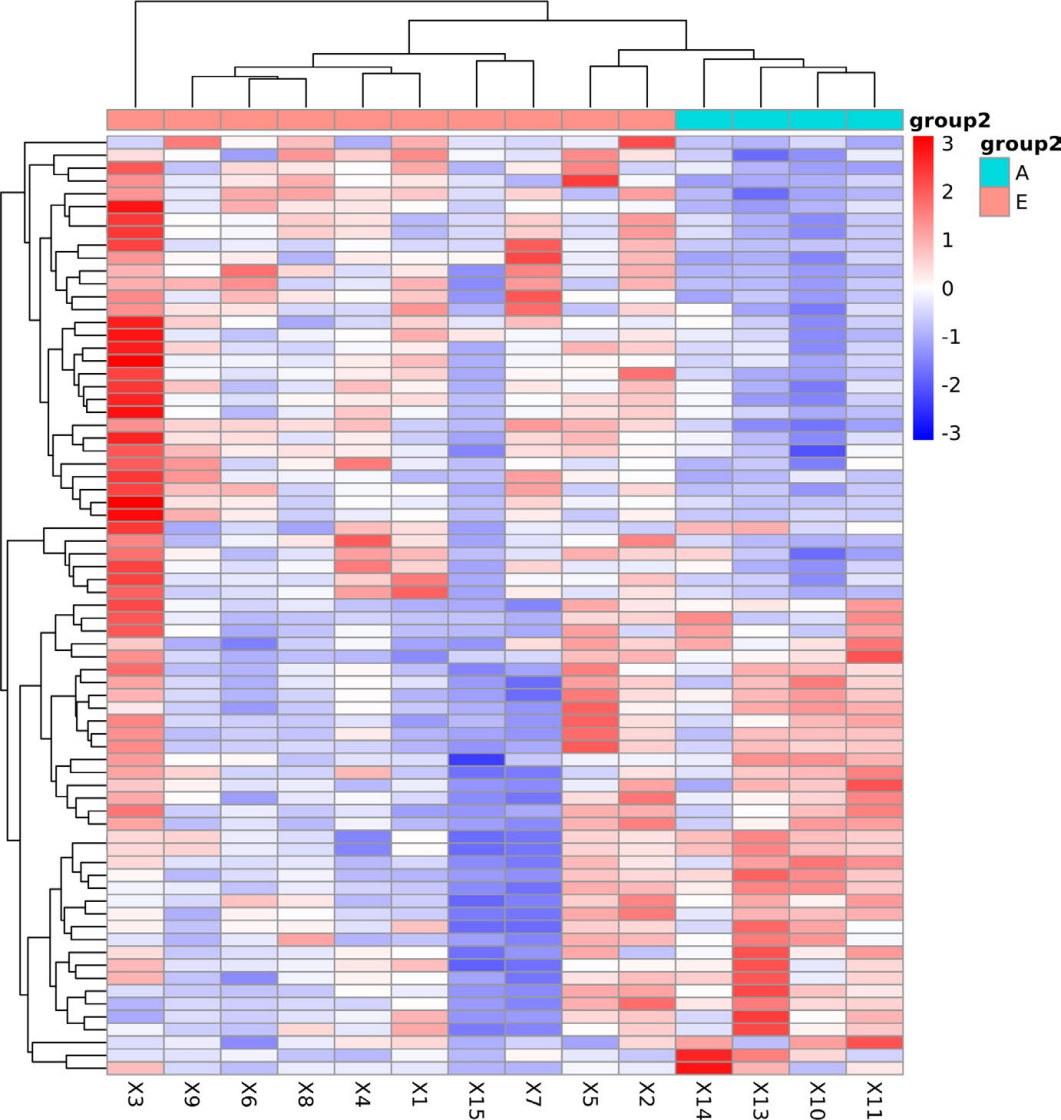
Differential gene expression between COVID‐19 patients and healthy donors. Each column represents a different sample. Lines represent different genes. The color represents the level of gene expression in the sample. Red indicates a high gene expression in the sample, and blue indicates a low gene expression in the sample

Hierarchical clustering analysis of differentially expressed miRNAs showed that the miRNAs with a similar expression pattern were clustered. As shown in Figure 3, there were significant differences in the miRNA expression between the COVID‐19 patients and the control group.

### 3.4 Enrichment analysis of differentially expressed miRNA target genes

**Figure 4:**
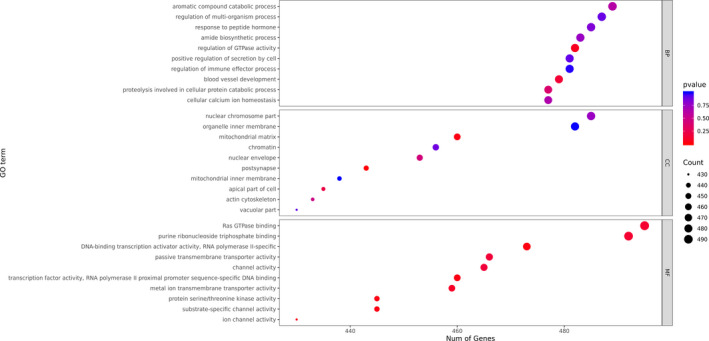
The GO classification of differentially expressed genes between the COVID‐19 patients and the control group

**Figure 5:**
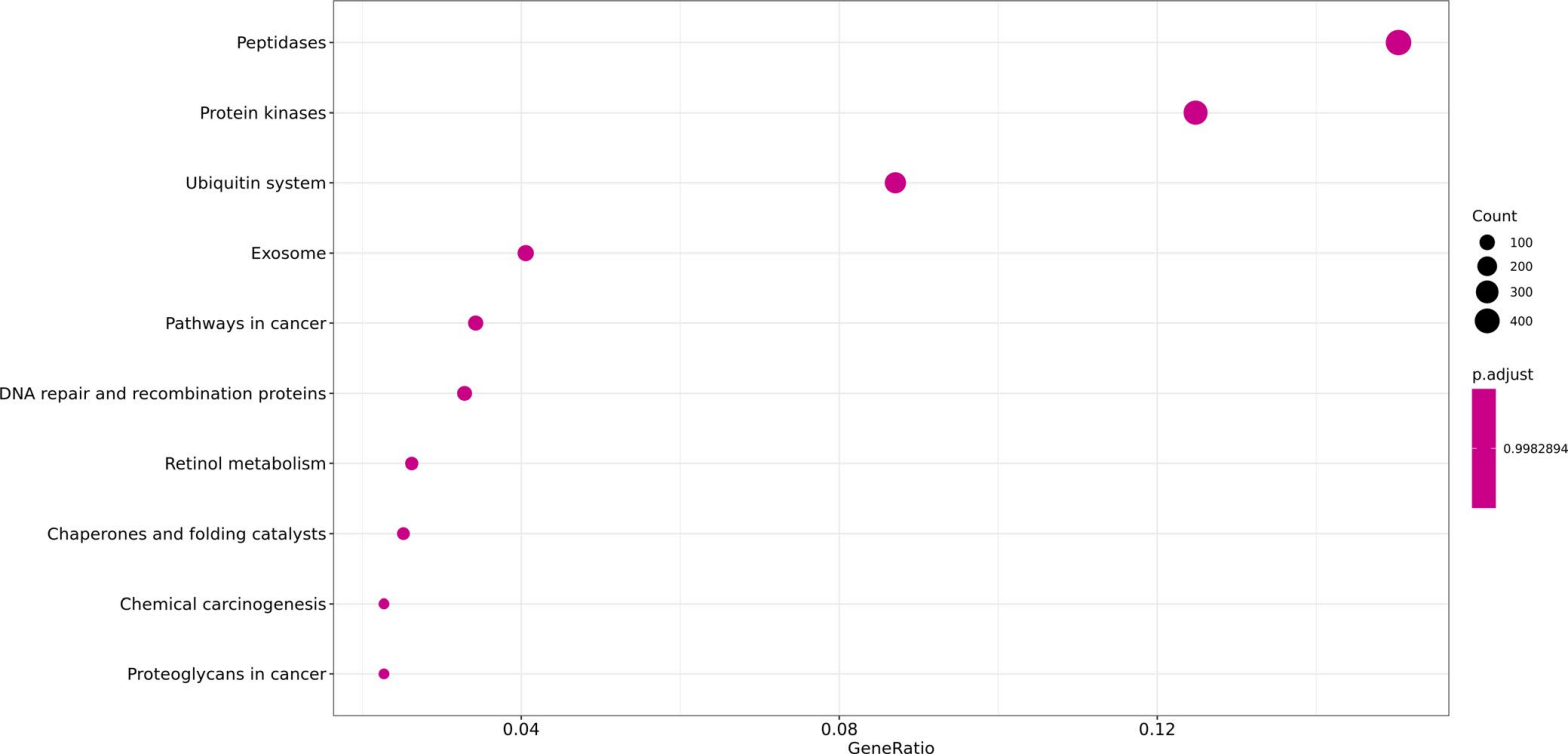
KEGG enrichment analysis of the differentially expressed genes

After screening the differentially expressed genes between the COVID‐19 patients and the healthy donors, the distribution of the GO of the differentially expressed genes was studied to predict their gene functions. The GO classification analysis, shown in Figure 4, suggested that the aromatic compound catabolic process, regulation of multi‐organism process, and response to peptide hormone were the most enriched biological processes with a significant difference between the two groups. In addition, the nuclear chromosome part, organelle inner membrane, and mitochondrial matrix were cellular compartments with significant differences between the two groups. Moreover, Ras GTPase binding, purine ribonucleoside, and purine ribonucleoside triphosphate binding were the most enriched molecular functions with a significant difference between the two groups. As shown in Figure 5, peptidases, protein kinases, and the ubiquitin system were the most enriched categories by KEGG enrichment.

## 4 DISCUSSION

In the present study, the differential miRNA expression in peripheral blood from COVID‐19 patients and healthy donors was observed. Clinically, cytokine release syndrome (CRS) is a common phenomenon in patients with COVID‐19.^4^ Activation of CRS results in pleiotropic effects on both the innate and acquired immune systems. For example, corticosteroids are often administered in sepsis‐associated acute respiratory distress syndrome patients; in addition, clinical data support the use of corticosteroid treatment for lung injury in COVID‐19 patients.^17^ However, inhibiting inflammatory cytokines or providing T‐cell therapy is not a good option for treating patients with COVID‐19.^4^

MiRNAs play very important roles in modulating the immune response during viral infections. MiRNAs can repress gene expression by targeting host cellular RNAs or viral RNAs during infection.^13^, ^14^, ^15^ Furthermore, miRNAs have essential functions in a number of immune‐related diseases.^11^, ^18^ For example, mechanistically, aberrant expression of miRNAs can contribute to the Th17/Treg imbalance in immune thrombocytopenic purpura patients; this finding may offer clues regarding potential targets for novel treatments.^11^ The association between miRNAs and COVID‐19 remains to be investigated. Our data suggest that miR‐16‐2‐3p, miR‐6501‐5p, and miR‐618 were more highly expressed in COVID‐19 patients than in healthy controls and that miR‐183‐5p, miR‐627‐5p, and miR‐144‐3p were less expressed in COVID‐19 patients than in healthy controls. Several studies have found that miRNA‐618 differentially expressed, which was similar in our study, is related to dysregulation of immune function.^12^, ^18^, ^19^, ^20^ Moreover, miRNA‐618 expression has been shown to be upregulated in plasmacytoid dendritic cells (pDCs) in systemic sclerosis patients. Furthermore, it has been shown that the upregulation of miR‐618 also suppresses the development of pDCs and increases their ability to secrete interferon‐alpha.^12^ In addition, miRNA‐618 directly targets metadherin mRNA to suppress the malignant phenotype of osteosarcoma cells by reducing phosphatase and tensin homolog (PTEN)/protein kinase B (AKT) pathway signaling.^20^

Our data indicate that the levels of Ras GTPase binding and protein kinases were significantly different between the COVID‐19 patients and healthy controls. The GTPases of the Ras superfamily can regulate cell growth, membrane trafficking, and the cytoskeleton *in vivo*. Additionally, miRNAs have been found to participate in the regulation of GTPases in many tumor studies. Dysregulation of miR‐618 also is involved in prostate cancer and thyroid carcinoma.^20^ Interestingly, it has been shown that miR‐618 reduces the PTEN‐AKT pathway output in osteosarcoma cells both *in vitro* and *in vivo*.^20^ Similarly, miRNAs have been implicated in the regulation of other protein kinases.^18^ Nonetheless, how the expression of miR‐618 is regulated in COVID‐19 patients still remains unclear and requires further investigation.

Nevertheless, several limitations existed in this study. First, the specimens were obviously collected at different times for the infectivity of specimens could not be collected at the time of diagnosis.

## 5 CONCLUSION

To the best of our knowledge, for the first time, our data reveal the dysregulated expression of miRNAs in the peripheral blood of COVID‐19 patients. Thus, miR‐618 may be a promising therapeutic and diagnostic target to treat COVID‐19 patients.

## ETHICAL APPROVAL

This study was approved by the Ethics Committee of the Fourth Affiliated Hospital, College of Medicine, Zhejiang University. All subjects were asked to sign formal consents prior to their enrollment in this study. This study was registered at the Chinese Clinical Trial Registry, ChiCTR2000030305 (http://www.medresman.org.cn/login.aspx).
